# Association between Dietary Vitamin E Intake and Human Papillomavirus Infection among US Adults: A Cross-Sectional Study from National Health and Nutrition Examination Survey

**DOI:** 10.3390/nu15173825

**Published:** 2023-09-01

**Authors:** Qian Zhou, Mengjiao Fan, Yanrong Wang, Yue Ma, Haiyan Si, Guanghai Dai

**Affiliations:** 1 Department of Oncology, Medical School of Chinese People’s Liberation Army, Beijing 100853, China; qianzhouwhu@163.com (Q.Z.); fanmengjiao1201@163.com (M.F.); bellasince1987@163.com (Y.W.); 301oncmy@sina.com (Y.M.); 2 The Fifth Medical Center, Department of Medical Oncology, Chinese People’s Liberation Army General Hospital, Beijing 100853, China; sihaiyan2008@sina.com

**Keywords:** nutritional epidemiology, vitamin E, human papillomavirus, cervical cancer, penile cancer, oropharyngeal cancer

## Abstract

Persistent high-risk human papillomavirus (HPV) infection is responsible for most genital, anal, and oropharyngeal cancers, in which men contribute significantly to infection and subsequent tumorigenesis in women. Vitamin E has been shown to be associated with vaginal HPV infection and cervical cancer. However, the association of vitamin E consumption with HPV infection among the overall population remains unclear. We investigate the association between vitamin E consumption and genital and oral HPV infection in both men and women. We used cross-sectional data from the National Health and Nutrition Examination Survey between 2013 and 2016 to collect details on their dietary vitamin E intake, genital and oral HPV infection status, and other essential variables. In total, 5809 participants aged 18–59 years were identified, with overall prevalence of high-risk and low-risk HPV infection of 23.7% and 21.1%, respectively. Compared with the lowest vitamin E group Q1 (<5.18 mg/day), the adjusted OR for vitamin E consumption and overall high-risk HPV infection in Q2 (5.18–7.54 mg/day), Q3 (7.55–10.82 mg/day), and Q4 (>10.82 mg/day) were 0.91 (95% CI: 0.81–1.03, *p* = 0.134), 0.77 (95% CI: 0.69–0.87, *p* < 0.001), and 0.72 (95% CI: 0.65–0.80, *p* < 0.001), respectively. Restricted cubic spline regression showed a linear relationship between vitamin E consumption and overall high-risk HPV infection. This linear relationship also existed for vitamin E consumption and overall low-risk HPV infection. After being stratified by gender and site, vitamin E consumption was inversely related to vaginal low- and high-risk HPV infection, penile high-risk HPV infection, and male oral low-risk HPV infection. In conclusion, we identified inverse linear relationships between dietary vitamin E intake and overall high- and low-risk HPV infection. Future well-designed longitudinal studies are still required to validate the impact of vitamin E on HPV carcinogenesis.

## 1. Introduction

Human papillomavirus (HPV) is one of the most ubiquitous sexually transmitted viruses around the world, which is usually asymptomatic, temporary, and eliminated by their hosts’ immune system within 1–2 years [1]. Nevertheless, the persistent infection of certain HPV strains, known as oncogenic or high-risk HPV, can lead to a preneoplastic lesion and ultimately cause cancer if left unchecked [2,3]. Nearly all cervical cancers, 90% of squamous anal cancers, 50% of vulvar and penile cancers, and some head and neck cancers are brought on by persistent high-risk HPV infection in the corresponding site [3,4,5,6,7]. HPV infection is widespread among sexually active people, and male HPV infection significantly contributes to female HPV infection and consequent diseases [8]. Although immunization and safe sex education are efficient approaches to reducing high-risk HPV prevalence, effective HPV vaccination and screening programs are still difficult to execute worldwide. Even in the US, the current HPV vaccination rate is low [9,10]. Therefore, it is crucial to investigate other possible HPV infection-related tactics that could help with either prevention or treatment.

There is growing evidence that consuming “healthy” foods such as fruits and vegetables and taking in specific nutrients such as antioxidants and minerals can reduce the risk of contracting HPV, developing preneoplastic lesions, and subsequent cervical cancer among women [11,12,13,14,15,16,17,18]. Cell signaling and homeostasis in response to infections are highly reliant on the cellular oxidative environment and the formation of reactive oxygen species (ROS) [19]. Previous studies indicated that the risk of HPV infection is inversely related to antioxidant plasma concentrations [20,21,22]. As one of the most important antioxidants, vitamin E has been confirmed to prevent DNA damage, inhibit cell proliferation, and enhance immune functions [8,14]. Because it can promote inflammatory reactions and inhibit the replication of HPV, vitamin E consumption has been found to reduce the incidence of cervical cancer and persistent HPV infection in women [23]. In addition, vitamin E consumption and plasma vitamin E concentration had a negative correlation with the incidence of cervical neoplasia [24,25]. However, the current studies into the relationship between dietary vitamin E intake and HPV infection remain limited, especially in gender-specific populations. A secondary analysis utilizing data from the National Health and Nutrition Examination Survey (NHANES) revealed a significant inverse relationship between dietary vitamin E intake and vaginal high-risk HPV infection in women [15]. In contrast, another global prospective study examining the natural course of penile HPV infection found no correlation between vitamin E consumption and persistent HPV infection in men [8]. Given the critical transmission role of men in female HPV infection, we believe it is necessary to include different sex populations in one study, but this has not been demonstrated to date. In addition, current research on vitamin E consumption and HPV infection has only focused on reproductive tract infections. Oral sex is one of the crucial ways of sexual transmission, and the relationship between oral HPV infection and vitamin E consumption has not been evaluated.

To fill these research gaps, we investigated the association of dietary vitamin E intake with genital and oral HPV infection in both men and women, especially high-risk HPV infection among US adults, with data from the NHANES. As a substitute for cervical HPV infection in this investigation, we collected data on vaginal HPV infection. Prior research revealed an excellent consistency of the detection of HPV between cervical swabs obtained from clinicians and vaginal swabs obtained from their own [26,27]. Accordingly, we hypothesized an inverse association of vitamin E consumption with overall HPV infection. Next, we evaluated the differences in gender and site of the association between vitamin E consumption and HPV infection. The dose-response relationship between vitamin E consumption and overall HPV infection was also assessed.

## 2. Materials and Methods

### 2.1. Data Source and Study Population

Data from the NHANES, a US nationally representative survey conducted by the National Center for Health Statistics (NCHS), using stratified, multistage probability cluster sampling to track the nutritional status of the US population who are not institutionalized, were used in this cross-sectional study. The details of NHANES are available on the official website [28]. The NHANES study protocol was approved by the NCHS Ethics Review Committee, and all individuals signed written informed consent at enrollment. Because this study is a secondary analysis using publicly available deidentified data, the ethics statements are waived, and the informed consent is not applicable. This study included NHANES participants from 2013 to 2016 because male penile HPV infection data are only available on NHANES from 2013 to 2016. Participants under 18 or over 59 were excluded because the age range for detecting genital HPV infection is 18–59. Participants with missing data on genital HPV infection, oral HPV infection, and dietary vitamin E intake were excluded. Additionally, those who had received any dosage of the HPV vaccine were not included in this study.

### 2.2. Detection and Classification of HPV Infections

A total of 37 HPV genotypes (6, 11, 16, 18, 26, 31, 33, 35, 39, 40, 42, 45, 51, 52, 53, 54, 55, 56, 58, 59, 61, 62, 64, 66, 67, 68, 69, 70, 71, 72, 73, 81, 82, 83, 84, 89, and IS39) were detected in NHANES with the Linear Array HPV Genotyping Assay (Roche Diagnostic) on samples from self-collected vaginal or penile swabs and oral rinse. This assay uses biotinylated PGMY09/11 primer sets in HPV L1 consensus polymerase chain reaction (PCR). More details of the testing procedures are available on the Laboratory Data section of the NHANES website [28]. The outcome variable in this study was overall HPV infection status, which was categorized as negative (negative for all 37 HPV types at genital and oral sites), high-risk HPV infection [at least one of the 14 oncogenic HPV types (16, 18, 31, 33, 35, 39, 45, 51, 52, 56, 58, 59, 66, and 68) positive at genital or oral sites], and low-risk HPV infection (at least one of the remaining 23 non-oncogenic HPV types positive and no oncogenic HPV infection at genital or oral sites).

### 2.3. Assessment of Dietary Vitamin E Intake and Covariates

Participants in the NHANES were asked to calculate their dietary consumption in detail, including the calorie intake, nutrients, and other components of food they consumed in the 24 h before the interview. Everyone who took part in the NHANES was qualified for two 24 h dietary recall interviews. In the Mobile Examination Center (MEC), the initial dietary recall interview was conducted in person, and the second interview was conducted over the phone three to ten days later. The NHANES dietary interviewer procedures manuals comprehensively document the examination protocol and data-gathering techniques [29]. In our study, the 24 h dietary vitamin E intake was calculated from the average of the two 24 h dietary recall interviews.

According to previous literature [8,15,16,18], our study considered potential confounders such as age, sex, race, education, marital status, number of persons in the household, family income, smoking, sleep hours, age at first sexual intercourse, number of sexual intercourses past year, number of sex partners during the lifetime, illegal substance use, alcohol consumption, body mass index (BMI), and calorie consumption. In the female population, the number of pregnancies and oral contraceptive use were also considered. The detailed classification of these covariates is presented in Table 1. Family income was divided into three groups based on the poverty income ratio (PIR): low (PIR ≤ 1.3), medium (1.3 < PIR ≤ 3.5), and high (PIR > 3.5) [30]. We determined whether a participant was a never smoker, current smoker, or former smoker based on their replies to two questions: “have you smoked at least 100 cigarettes in your entire life?” and “do you now smoke cigarettes?”. Illegal substance refers to marijuana or hashish. Calorie consumption was estimated based on the average of two 24 h dietary recall interviews.

### 2.4. Statistical Analyses

Participants’ baseline characteristics were expressed as means with standard deviations (SD) (normal distribution) or medians with interquartile ranges (IQR) (skewed distribution) for continuous variables and as frequencies with percentages (%) for categorical variables. The participants were split into four groups (Q1, Q2, Q3, and Q4) based on the dietary vitamin E intake quartile. For continuous variables, the differences in baseline characteristics between four groups were examined using one-way ANOVA (normal distribution) or the Kruskal-Wallis test (skewed distribution), and for categorical variables, the chi-square test.

To replace missing covariate data with statistical estimates, we used dummy variables to indicate missing continuous covariate values and 9 was used as a level for missing categorical covariates. We also performed sensitivity analyses using a complete data analysis. The correlation between covariates and HPV infection status was investigated using univariate multinomial logistic regression. To evaluate the relationship between dietary vitamin E intake and HPV infection status, we used multivariate multinomial logistic regression models. The odds ratios (ORs) and 95% confidence intervals (CIs) were calculated. We conducted three models: the crude model did not adjust any confounders; Model 1 was modified to account for sociodemographic factors such as age, sex, race, education, marital status, number of persons in the household, and family income; Model 2 was the fully adjusted model, which included adjustments for all of the factors shown in Table 2. In order to confirm the findings of dietary vitamin E as the continuous variable and to test for nonlinearity, we converted dietary vitamin E into a categorical variable according to the quartile and calculated the *p* for trend.

To further explore nonlinearity, restricted cubic spline (RCS) regression was conducted with four knots located at the 5th, 35th, 65th, and 95th percentiles of dietary vitamin E intake to look at the dose-response relationship between vitamin E consumption and HPV infection status.

In order to examine the differences in gender and site of the relationship between vitamin E consumption and HPV infection status, stratified analysis was performed, including samples from female vaginal swabs, female oral rinses, male penile swabs, and male oral rinses.

Additionally, we conducted sensitivity analysis by removing those with extreme calorie consumption (less than 500 or more than 5000 kcal per day) and missing covariate values in multivariate multinomial logistic regression models to evaluate the robustness of the results.

All statistical analyses were performed with R, version 4.3.1 (http://www.R-project.org, The R Foundation, Shanghai, China) (accessed on 1 March 2023) and Free Statistics, version 1.7.1. *p*-value < 0.05 (two-sided) was declared statistically significant.

## 3. Results

### 3.1. Population Characteristics

**Figure 1:**
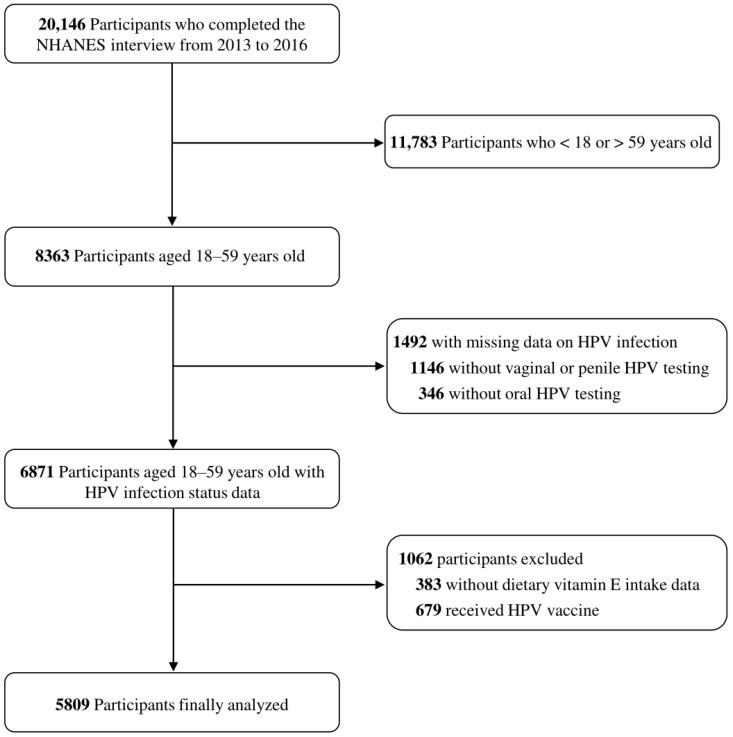
The flow diagram of this study. Abbreviations: NHANES, National Health and Nutrition Examination Survey; HPV, human papillomavirus.

A total of 20,146 participants completed the NHANES interview from 2013 to 2016, of whom 11,783 participants were under 18 or over 59 years old. We excluded 1146 participants with missing data on genital HPV testing, 346 participants with missing data on oral HPV testing, 383 participants with missing data on dietary vitamin E intake, and 679 participants with any dose of HPV vaccine. Ultimately, this study included 5809 participants in total. The details of the inclusion and exclusion procedure are presented in Figure 1. In Supplementary Table S1, the baseline characteristics of the included and excluded subjects are displayed.

The overall prevalence of high-risk and low-risk HPV infection was 23.7% (1375/5809) and 21.2% (1234/5809), respectively. The sociodemographic and behavioral characteristics of vitamin E intake quartiles are shown in Table 1. The average age of the population included in the study was 39.1 (11.9) years, and half were male (2907/5809). Participants with higher levels of vitamin E intake tended to be male, non-Hispanic white, more educated, living in a couple, had a higher family income, former smokers, had the first sex later in life, had more sex partners, illegal substance use, more alcohol consumption, lower BMI, more calorie consumption, and no HPV infection.

### 3.2. Association between Dietary Vitamin E Intake and HPV Infection

As presented in Table 2, sex, race, education, marital status, number of persons in the household, family income, smoking status, sleep hours, age at first sexual intercourse, number of sex partners during the lifetime, illegal substance use, and calorie consumption were associated with overall high-risk HPV infection. These factors were also associated with overall low-risk HPV infection, except for sex and alcohol consumption.

We conducted three models to examine the association of dietary vitamin E intake with HPV infection status. In the unadjusted and sociodemographic adjusted models, for every 1 mg increase of dietary vitamin E consumption per day, the risk of overall high-risk HPV infection decreased by 1% (OR = 0.99, *p* < 0.05). This inverse relationship between vitamin E consumption and overall high-risk HPV infection was even more pronounced in the fully adjusted model (OR = 0.98, 95% CI: 0.97–0.99, *p* = 0.004). The inverse relationship was also found in vitamin E consumption and overall low-risk HPV infection, with an estimated OR value of 0.99 (*p* < 0.05) in the unadjusted model and sociodemographic adjusted model and an estimated OR value of 0.98 (95% CI: 0.97–1.00, *p* = 0.021) in the fully adjusted model (Table 3).

**Figure 2:**
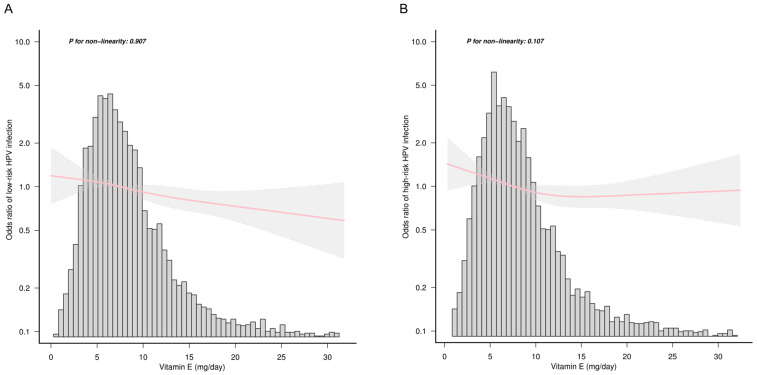
Association of dietary vitamin E intake with (**A**) overall low-risk and (**B**) high-risk HPV infection. The solid pink lines and dotted pink lines represent the predicted odds ratios and 95% confidence intervals, respectively. All covariates presented in Table 2 were adjusted. Only the top 99% of dietary vitamin E intake data were shown.

To test the likelihood of a nonlinear association between dietary vitamin E intake and HPV infection, we converted vitamin E into a categorical variable based on the quartile. Compared with the lowest vitamin E group Q1 (<5.18 mg/day), the fully adjusted OR values for vitamin E consumption and overall high-risk HPV infection in Q2 (5.18–7.54 mg/day), Q3 (7.55–10.82 mg/day), and Q4 (>10.82 mg/day) were 0.91 (95% CI: 0.81–1.03, *p* = 0.134), 0.77 (95% CI: 0.69–0.87, *p* < 0.001), and 0.72 (95% CI: 0.65–0.80, *p* < 0.001), respectively. The result of the trend test suggested a linear relationship between dietary vitamin E intake and overall high-risk HPV infection (*p* for trend = 0.002). Compared with the lowest vitamin E group Q1, the fully adjusted OR values for vitamin E consumption and overall low-risk HPV infection in Q2, Q3, and Q4 were 0.96 (95% CI: 0.84–1.08, *p* = 0.475), 0.89 (95% CI: 0.79–1.00, *p* = 0.042), and 0.75 (95% CI: 0.68–0.84, *p* < 0.001), respectively. The trend test also suggested a linear relationship between dietary vitamin E intake and overall low-risk HPV infection (*p* for trend = 0.018) (Table 3). Furthermore, the linear relationship between dietary vitamin E intake and overall high- and low-risk HPV infection was confirmed by RCS (*p* for nonlinearity > 0.05) (Figure 2).

### 3.3. Stratified Analysis by Gender and Site

**Figure 3:**
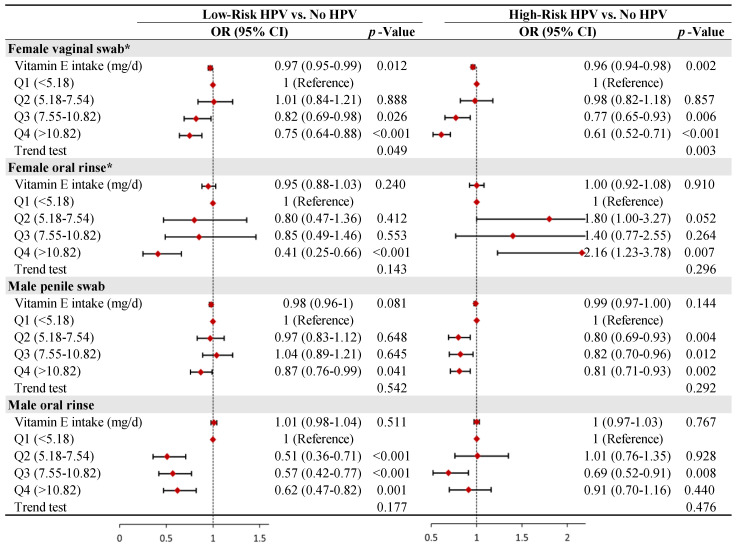
Gender– and site–-stratified analysis of the association between dietary vitamin E and HPV infection. Each stratification was adjusted for all covariates presented in Table 2, except for sex. * Number of pregnancies and oral contraceptive use were additionally adjusted in the female population. Only the top 99% of dietary vitamin E intake data were shown.

A stratified analysis was conducted to investigate the relationship between dietary vitamin E intake and HPV infection in participants with different genders and infection sites. As shown in Figure 3, in the female population, the inverse linear relationship between dietary vitamin E intake and vaginal high- and low-risk HPV infection (*p* for trend < 0.05) remained stable. In the male population, dietary vitamin E intake was inversely but nonlinearly correlated with penile high-risk HPV infection (*p* for trend = 0.292) and was not significantly correlated with penile low-risk HPV infection. As for the risk of oral HPV infection, dietary vitamin E intake was only inversely correlated with male oral low-risk HPV infection and was not significantly correlated with male oral high-risk HPV infection and female oral HPV infection.

### 3.4. Sensitivity Analysis

After excluding 86 participants with extreme calorie consumption, the inverse linear relationship between dietary vitamin E intake and overall HPV infection remained stable (Supplementary Table S2). Compared with the lowest vitamin E group Q1, the fully adjusted OR values for vitamin E intake and overall high-risk HPV infection in Q2, Q3, and Q4 were 0.93 (95% CI: 0.82–1.06, *p* = 0.270), 0.80 (95% CI: 0.71–0.89, *p* < 0.001), and 0.74 (95% CI: 0.67–0.82, *p* < 0.001), respectively, and the fully adjusted OR values for overall low-risk HPV infection in Q2, Q3, and Q4 were 0.94 (95% CI: 0.83–1.06, *p* = 0.330), 0.86 (95% CI: 0.77–0.97, *p* = 0.012), and 0.73 (95% CI: 0.66–0.81, *p* < 0.001), respectively. After excluding 2253 participants with incomplete covariate data, the inverse linear association between dietary vitamin E intake and overall high-risk HPV infection remained stable (Supplementary Table S3).

## 4. Discussion

Persistent high-risk HPV infection is a prerequisite for developing cervical, anal, penile, and oropharyngeal cancers [3,4,5,6,7]. Previous studies have demonstrated how diets and nutrients might influence HPV infection and cervical cancer among women [11,12,13,14,15,16,17,18]. Vitamin E has been investigated as a potential moderator of HPV infection and cervical cancer in women in earlier research [15,23,24,25]. To our knowledge, no studies have reported the association between dietary vitamin E intake and HPV infection risk in the overall population of both men and women at genital and oral sites. Our extensive cross-sectional investigation of adult Americans revealed an inverse linear relationship between vitamin E consumption and overall high- and low-risk HPV infection. The stratified analysis confirmed that dietary vitamin E intake is inversely associated with high-risk HPV infection in the vaginal and penile sites. However, neither in men nor in women was there a significant association between dietary vitamin E intake and oral high-risk HPV infection.

It is well-known that host factors play a crucial role in acquiring and preventing HPV infection. These host factors can be divided into behavioral and biological [31]. Behavioral risk factors, such as sexual conduct, influence the acquisition of HPV and have been adjusted in Model 2 in this study. Biological risk factors mainly affect the persistence of HPV and the development of cancers by impacting immunologic function [32]. Nutritional status has been shown to be critical in supporting the immune system’s regular activities. Nutrient deficiencies usually lead to impaired immunologic function, and conversely, intakes at or beyond suggested levels can restore or further improve immunologic function [33]. Vitamin E is one of the most effective nutrients for regulating immunologic function. Immune cells have a higher vitamin E concentration than other blood cells because vitamin E is a major lipid-soluble antioxidant that scavenges ROS and reduces the oxidation of polyunsaturated fatty acids (PUFAs), which are abundant in immune cell membranes [34].

Although the exact mechanism causing the inverse correlation between vitamin E consumption and HPV infection remains to be researched, our findings seem biologically tenable considering the current evidence on the immunomodulatory effects of vitamin E. First, as previously demonstrated, immune cells have an exceptionally high vitamin E content, which may help to preserve the high PUFAs content of their membranes from oxidative damage caused by their high metabolic activities and normal defense function [34,35]. Immune cells rely heavily on the composition and structure of their cell membranes since they serve as the primary site where external signals are transferred to the plasma and nucleus to regulate essential genes through various signal transduction mechanisms. By reducing lipid peroxidation and the related cell membrane damage, vitamin E may help preserve cell membrane integrity, sustain signal transduction and the generation of essential proteins and other mediators, and directly strengthen the immune system [36]. Second, prostaglandin E2 (PGE2) has been reported to impact both the innate and adaptive immune systems by decreasing T cell proliferation, IL-2 receptor expression, and IL-2 production [37,38,39]. By suppressing the enzyme activity of cyclooxygenase 2 (COX-2), a rate-limiting enzyme involved in the conversion of arachidonic acid to prostaglandins, vitamin E supplementation may reduce PGE2 synthesis and exerts indirect regulatory effects on the immune system [40]. When taken together, vitamin E protects immune cell membrane integrity, suppresses PGE2 generation, and has significant antioxidant effects, which may be the molecular mechanism underpinning high vitamin E intake for HPV prevention.

Our research broadens the comprehension of the relationship between dietary vitamin E intake and HPV infection risk in the overall population at genital and oral sites. Our findings suggest that vitamin E deficiency, which is thought to be related to immune dysregulation, might have a more significant impact on genital HPV infection than oral HPV infection in both men and women and might indicate that HPV has a different tendency for clearance and persistence in different sites. Our results imply that human reproductive mucosa might be more vulnerable to vitamin E’s immunomodulatory effects. These findings, if supported by prospective studies of the association between diet vitamin E intake and HPV persistence, might inform novel dietary-based preventive strategies against genital high-risk HPV infection, particularly in areas where male vaccination programs have not yet been implemented, or vaccination coverage levels are inadequate. Given that it takes decades for persistent high-risk HPV infection to progress to cancers, this lengthy window offers a perfect chance for clinical intervention.

## 5. Limitations

Several limitations should be considered when interpreting these results. First, the NHANES only collected information on male penile HPV from 2013 to 2016, which stopped us from further validating these findings using NHANES data from other periods. Second, the 24 h recall was used to determine vitamin E consumption, which could introduce recall bias. However, compared to the 24 h recall, the food frequency survey offers less accurate data on food types and quantities [41,42]. Third, because only a single HPV infection was assessed, this study cannot determine the relationship between vitamin E intake and HPV persistence, and further prospective studies are required to understand this relation better. Fourth, only dietary vitamin E intake was collected in this study, and vitamin E intake from supplements was missing from the NHANES from 2013 to 2016, which could be an important confounding factor. Therefore, the data on vitamin E intake from supplements should be included in future studies. Finally, the causal relationship between vitamin E consumption and HPV infection cannot be deduced due to the intrinsic limitation of cross-sectional NHANES data and must be further validated by longitudinal research in the future.

## 6. Conclusions

In conclusion, our study demonstrated inverse linear relationships between dietary vitamin E intake and overall high- and low-risk HPV infection. For both men and women, vitamin E may have a preventive effect on genital high-risk HPV infection. The findings of this study highlight the link between diet-related vitamin E consumption and HPV infection. Future well-designed longitudinal studies are still required to validate the impact of vitamin E on HPV carcinogenesis.

## Figures and Tables

**Table 1:** Characteristics of 5809 participants by categories of dietary vitamin E intake.

Characteristic	Dietary Vitamin E Intake, mg/d					
	Total	Q1 (<5.18)	Q2 (5.18–7.54)	Q3 (7.55–10.82)	Q4 (>10.82)	*p*-Value
No.	5809	1452	1452	1450	1455	
Age (years), mean (SD)	39.1 (11.9)	38.8 (12.3)	38.9 (11.9)	39.4 (11.8)	39.2 (11.6)	0.448
Sex, *n* (%)						<0.001
Male	2907 (50.0)	616 (42.4)	683 (47)	773 (53.3)	835 (57.4)	
Female	2902 (50.0)	836 (57.6)	769 (53)	677 (46.7)	620 (42.6)	
Race, *n* (%)						<0.001
Non-Hispanic white	2081 (35.8)	471 (32.4)	498 (34.3)	534 (36.8)	578 (39.7)	
Non-Hispanic black	1207 (20.8)	304 (20.9)	320 (22)	281 (19.4)	302 (20.8)	
Mexican American	978 (16.8)	241 (16.6)	260 (17.9)	254 (17.5)	223 (15.3)	
Others	1543 (26.6)	436 (30)	374 (25.8)	381 (26.3)	352 (24.2)	
Education level (years), *n* (%)						<0.001
<9	365 (6.3)	137 (9.4)	97 (6.7)	84 (5.8)	47 (3.2)	
9–12	1939 (33.4)	591 (40.7)	487 (33.5)	462 (31.9)	399 (27.4)	
>12	3189 (54.9)	635 (43.7)	773 (53.2)	828 (57.1)	953 (65.5)	
NA	316 (5.4)	89 (6.1)	95 (6.5)	76 (5.2)	56 (3.8)	
Marital status, *n* (%)						<0.001
Living in a couple	3439 (59.2)	794 (54.7)	866 (59.6)	879 (60.6)	900 (61.9)	
Living alone	2054 (35.4)	569 (39.2)	491 (33.8)	494 (34.1)	500 (34.4)	
NA	316 (5.4)	89 (6.1)	95 (6.5)	77 (5.3)	55 (3.8)	
No. of persons in household, *n* (%)						0.003
1	490 (8.4)	110 (7.6)	115 (7.9)	131 (9)	134 (9.2)	
2–3	2369 (40.8)	577 (39.7)	589 (40.6)	599 (41.3)	604 (41.5)	
4–6	2508 (43.2)	616 (42.4)	646 (44.5)	614 (42.3)	632 (43.4)	
>6	442 (7.6)	149 (10.3)	102 (7.0)	106 (7.3)	85 (5.8)	
Family income, *n* (%)						<0.001
Low	1821 (31.3)	593 (40.8)	486 (33.5)	403 (27.8)	339 (23.3)	
Medium	1924 (33.1)	469 (32.3)	474 (32.6)	482 (33.2)	499 (34.3)	
High	1616 (27.8)	270 (18.6)	391 (26.9)	444 (30.6)	511 (35.1)	
NA	448 (7.7)	120 (8.3)	101 (7.0)	121 (8.3)	106 (7.3)	
Smoking status, *n* (%)						<0.001
Never	3532 (60.8)	830 (57.2)	917 (63.2)	900 (62.1)	885 (60.8)	
Current	1309 (22.5)	429 (29.5)	322 (22.2)	277 (19.1)	281 (19.3)	
Former	967 (16.6)	193 (13.3)	213 (14.7)	273 (18.8)	288 (19.8)	
NA	1 (0.0)	0 (0)	0 (0)	0 (0)	1 (0.1)	
Sleep hours, *n* (%)						0.051
<8	3476 (59.8)	852 (58.7)	877 (60.4)	868 (59.9)	879 (60.4)	
8–9	1944 (33.5)	476 (32.8)	470 (32.4)	502 (34.6)	496 (34.1)	
>9	372 (6.4)	117 (8.1)	101 (7)	76 (5.2)	78 (5.4)	
NA	17 (0.3)	7 (0.5)	4 (0.3)	4 (0.3)	2 (0.1)	
Age at first sexual intercourse (years), *n* (%)						0.016
Never	279 (4.8)	83 (5.7)	75 (5.2)	71 (4.9)	50 (3.4)	
<16	1545 (26.6)	397 (27.3)	368 (25.3)	399 (27.5)	381 (26.2)	
16–17	1551 (26.7)	383 (26.4)	384 (26.4)	381 (26.3)	403 (27.7)	
18–19	1034 (17.8)	246 (16.9)	287 (19.8)	247 (17.0)	254 (17.5)	
>19	1070 (18.4)	238 (16.4)	266 (18.3)	276 (19.0)	290 (19.9)	
NA	330 (5.7)	105 (7.2)	72 (5.0)	76 (5.2)	77 (5.3)	
No. of sexual intercourse past year, *n* (%)						<0.001
0	157 (2.7)	37 (2.5)	40 (2.8)	44 (3.0)	36 (2.5)	
1–11	1256 (21.6)	332 (22.9)	322 (22.2)	306 (21.1)	296 (20.3)	
12–51	1552 (26.7)	337 (23.2)	368 (25.3)	427 (29.4)	420 (28.9)	
52–103	923 (15.9)	200 (13.8)	223 (15.4)	235 (16.2)	265 (18.2)	
104–364	585 (10.1)	134 (9.2)	151 (10.4)	136 (9.4)	164 (11.3)	
365 or more	53 (0.9)	20 (1.4)	16 (1.1)	7 (0.5)	10 (0.7)	
NA	1283 (22.1)	392 (27)	332 (22.9)	295 (20.3)	264 (18.1)	
No. of sex partners during lifetime, *n* (%)						<0.001
0	327 (5.6)	90 (6.2)	86 (5.9)	81 (5.6)	70 (4.8)	
≤5	2593 (44.6)	681 (46.9)	664 (45.7)	643 (44.3)	605 (41.6)	
>5	2568 (44.2)	579 (39.9)	632 (43.5)	654 (45.1)	703 (48.3)	
NA	321 (5.5)	102 (7.0)	70 (4.8)	72 (5.0)	77 (5.3)	
Illegal substance use, *n* (%)						<0.001
Yes	2906 (50.0)	696 (47.9)	700 (48.2)	710 (49.0)	800 (55.0)	
No	2573 (44.3)	653 (45)	678 (46.7)	667 (46)	575 (39.5)	
NA	330 (5.7)	103 (7.1)	74 (5.1)	73 (5.0)	80 (5.5)	
No. of alcohol consumption past year, Median (IQR)	1.0 (0.0, 3.0)	1.0 (0.0, 3.0)	1.0 (0.0, 3.0)	1.5 (0.0, 3.0)	2.0 (0.0, 3.0)	<0.001
Body mass index (kg/m^2^), mean (SD)	29.2 (7.5)	29.3 (7.7)	29.6 (7.5)	29.1 (7.5)	28.8 (7.4)	0.035
Calorie consumption (kcal/day), Mean (SD)	2141.2 (901.0)	1451.4 (532.5)	1958.6 (562.1)	2313.1 (658.4)	2840.3 (1088.8)	<0.001
HPV infection status, *n* (%)						0.012
Negative	3200 (55.1)	748 (51.5)	785 (54.1)	822 (56.7)	845 (58.1)	
Low-risk HPV	1234 (21.2)	326 (22.5)	319 (22.0)	308 (21.2)	281 (19.3)	
High-risk HPV	1375 (23.7)	378 (26.0)	348 (24.0)	320 (22.1)	329 (22.6)	

Abbreviations: Q, quartiles; No., number; IQR, interquartile range; SD, standard deviation; NA, not available; HPV, human papillomavirus.

**Table 2:** Association of covariates with overall HPV infection.

Variables	Low-Risk HPV vs. No HPV		High-Risk HPV vs. No HPV	
	OR (95% CI)	*p*-Value	OR (95% CI)	*p*-Value
Age (years)	1.02 (1.01–1.02)	<0.001	1.00 (1.00–1.01)	0.454
Sex				
Male	1 (Reference)		1 (Reference)	
Female	0.92 (0.81–1.05)	0.238	0.68 (0.60–0.78)	<0.001
Race				
Non-Hispanic white	1 (Reference)		1 (Reference)	
Non-Hispanic black	2.50 (2.09–2.99)	<0.001	2.10 (1.76–2.49)	<0.001
Mexican American	0.92 (0.75–1.12)	0.398	0.75 (0.62–0.91)	0.004
Others	0.87 (0.73–1.04)	0.127	0.75 (0.64–0.89)	0.001
Education level (years)				
<9	1 (Reference)		1 (Reference)	
9–12	1.33 (1.01–1.77)	0.046	1.95 (1.46–2.60)	<0.001
>12	1.00 (0.76–1.31)	0.978	1.09 (0.82–1.45)	0.542
Marital status				
Living in a couple	1 (Reference)		1 (Reference)	
Living alone	1.65 (1.44–1.89)	<0.001	2.19 (1.91–2.50)	<0.001
No. of persons in household				
1	1 (Reference)		1 (Reference)	
2–3	0.84 (0.65–1.07)	0.163	0.63 (0.50–0.79)	<0.001
4–6	0.63 (0.49–0.80)	<0.001	0.47 (0.38–0.59)	<0.001
>6	0.64 (0.46–0.89)	0.008	0.49 (0.36–0.67)	<0.001
Family income				
Low	1 (Reference)		1 (Reference)	
Medium	0.92 (0.78–1.08)	0.310	0.83 (0.71–0.96)	0.015
High	0.68 (0.58–0.81)	<0.001	0.60 (0.51–0.71)	<0.001
Smoking status				
Never	1 (Reference)		1 (Reference)	
Current	2.24 (1.91–2.63)	<0.001	2.72 (2.34–3.18)	<0.001
Former	1.40 (1.17–1.68)	<0.001	1.51 (1.26–1.79)	<0.001
Sleep hours				
<8	1 (Reference)		1 (Reference)	
8–9	0.85 (0.73–0.98)	0.023	0.84 (0.73–0.96)	0.013
>9	0.79 (0.60–1.05)	0.110	0.96 (0.74–1.24)	0.728
Age at first sexual intercourse (years)				
Never	1 (Reference)		1 (Reference)	
<16	5.83 (3.79–8.96)	<0.001	8.99 (5.49–14.73)	<0.001
16–17	4.49 (2.92–6.91)	<0.001	7.16 (4.37–11.74)	<0.001
18–19	3.56 (2.29–5.53)	<0.001	5.28 (3.19–8.73)	<0.001
>19	1.86 (1.19–2.91)	0.007	2.84 (1.71–4.72)	<0.001
No. of sexual intercourse past year				
0	1 (Reference)		1 (Reference)	
1–11	1.22 (0.80–1.84)	0.356	1.28 (0.85–1.94)	0.234
12–51	0.90 (0.59–1.36)	0.607	1.00 (0.66–1.50)	0.983
52–103	0.88 (0.57–1.35)	0.564	1.08 (0.71–1.64)	0.727
104–364	1.21 (0.77–1.88)	0.408	1.52 (0.99–2.34)	0.058
365 or more	1.90 (0.90–4.00)	0.092	1.52 (0.70–3.30)	0.289
No. of sex partners during lifetime				
0	1 (Reference)		1 (Reference)	
≤5	2.04 (1.41–2.96)	<0.001	2.02 (1.37–2.98)	<0.001
>5	5.10 (3.52–7.38)	<0.001	7.01 (4.78–10.28)	<0.001
Illegal substance use				
Yes	1 (Reference)		1 (Reference)	
No	0.55 (0.48–0.63)	<0.001	0.43 (0.37–0.49)	<0.001
No. of alcohol consumption past year	1.01 (1.00–1.01)	0.058	1.01 (1.00–1.01)	0.070
Body mass index (kg/m^2^)	1.02 (1.01–1.02)	<0.001	1.00 (0.99–1.01)	0.955
Calorie consumption (kcal/day)	1.00 (1.00–1.00)	0.469	1.00 (1.00–1.00)	<0.001

Abbreviations: HPV, human papillomavirus; OR, odds ratio; CI, confidence interval; No., number.

**Table 3:** Association between dietary vitamin E intake and overall HPV infection.

	Crude ^a^		Model 1 ^b^		Model 2 ^c^	
	OR (95% CI)	*p*-Value	OR (95% CI)	*p*-Value	OR (95% CI)	*p*-Value
Low-Risk HPV vs. No HPV						
Vitamin E intake (mg/d)	0.99 (0.97–1.00)	0.010	0.99 (0.97–1.00)	0.016	0.98 (0.97–1.00)	0.021
Q1 (<5.18)	1 (Reference)		1 (Reference)		1 (Reference)	
Q2 (5.18–7.54)	0.93 (0.78–1.12)	0.456	0.96 (0.79–1.16)	0.665	0.96 (0.84–1.08)	0.475
Q3 (7.55–10.82)	0.86 (0.71–1.03)	0.109	0.89 (0.74–1.08)	0.238	0.89 (0.79–1.00)	0.042
Q4 (>10.82)	0.76 (0.63–0.92)	0.005	0.78 (0.64–0.95)	0.015	0.75 (0.68–0.84)	<0.001
Trend test		0.003		0.012		0.018
High-Risk HPV vs. No HPV						
Vitamin E intake (mg/d)	0.99 (0.98–1.00)	0.015	0.99 (0.97–1.00)	0.010	0.98 (0.97–0.99)	0.004
Q1 (<5.18)	1 (Reference)		1 (Reference)		1 (Reference)	
Q2 (5.18–7.54)	0.88 (0.74–1.05)	0.146	0.92 (0.77–1.11)	0.379	0.91 (0.81–1.03)	0.134
Q3 (7.55–10.82)	0.77 (0.64–0.92)	0.004	0.80 (0.66–0.97)	0.021	0.77 (0.69–0.87)	<0.001
Q4 (>10.82)	0.77 (0.65–0.92)	0.004	0.79 (0.65–0.95)	0.013	0.72 (0.65–0.80)	<0.001
Trend test		0.001		0.005		0.002

Abbreviations: HPV, human papillomavirus; Q, quartiles; OR, odds ratio; CI, confidence interval. ^a^ No covariates were adjusted in crude model. ^b^ Sociodemographic variables (age, sex, race, education, marital status, number of persons in household, and family income) were adjusted in Model 1. ^c^ All covariates presented in Table 2 (age, sex, race, education, marital status, number of persons in household, family income, smoking, sleep hours, age at first sexual intercourse, number of sexual intercourse past year, number of sex partners during lifetime, illegal substance use, alcohol consumption, body mass index, and calorie consumption) were adjusted in Model 2.

## Data Availability

Publicly available datasets are available online for this study. The repository/repositories name and accession numbers are available online at http://www.cdc.gov/nchs/nhanes.htm (accessed on 1 March 2023).

## Supplementary Materials

The following supporting information can be downloaded at: https://www.mdpi.com/article/10.3390/nu15173825/s1, Supplementary Table S1: Comparison of baseline characteristics between included and excluded populations; Supplementary Table S2: Sensitivity analysis of association between dietary vitamin E intake and overall HPV infection after excluding participants with extreme calorie consumption; Supplementary Table S3: Association between dietary vitamin E intake and overall HPV infection in participants with complete data.
