# Supplementary material for: Association between Dietary Vitamin E Intake and Human Papillomavirus Infection among US Adults: A Cross-Sectional Study from National Health and Nutrition Examination Survey
Source: Nutrients. 2023 Sep 1;15(17):3825. doi: 10.3390/nu15173825 (PMC10490162; doi:10.3390/nu15173825)
Supplement: Supplementary file 1 [file nutrients-15-03825-s001.zip › nutrients-2571540-supplementary.pdf]

Table S1. Comparison of baseline characteristics between included and excluded populations.

Characteristic	Total (n = 20146)	Included (n = 5809)	Excluded (n = 14337)	*p*-Value
Age (years), Mean (SD)	31.7 (24.6)	39.1 (11.9)	28.7 (27.6)	< 0.001
Sex, n (%)				0.094
Male	9895 (49.1)	2907 (50.0)	6988 (48.7)	
Female	10251 (50.9)	2902 (50.0)	7349 (51.3)	
Race, n (%)				< 0.001
Non-Hispanic white	6740 (33.5)	2081 (35.8)	4659 (32.5)	
Non-Hispanic black	4396 (21.8)	1207 (20.8)	3189 (22.2)	
Mexican American	3651 (18.1)	978 (16.8)	2673 (18.6)	
Others	5359 (26.6)	1543 (26.6)	3816 (26.6)	
Education level (years), n (%)				< 0.001
<9	1143 (10.0)	365 (6.6)	778 (13.0)	
9-12	4006 (34.9)	1939 (35.3)	2067 (34.5)	
>12	6327 (55.1)	3189 (58.1)	3138 (52.4)	
Marital status, n (%)				< 0.001
Living in a couple	6823 (59.4)	3439 (62.6)	3384 (56.5)	
Living alone	4659 (40.6)	2054 (37.4)	2605 (43.5)	
No. of persons in household, n (%)				< 0.001
1	1645 (8.2)	490 (8.4)	1155 (8.1)	
2-3	7008 (34.8)	2369 (40.8)	4639 (32.4)	
4-6	9593 (47.6)	2508 (43.2)	7085 (49.4)	
>6	1900 (9.4)	442 (7.6)	1458 (10.2)	
Family income, n (%)				< 0.001
Low	7100 (38.8)	1821 (34.0)	5279 (40.8)	
Medium	6562 (35.8)	1924 (35.9)	4638 (35.8)	
High	4647 (25.4)	1616 (30.1)	3031 (23.4)	
Smoking status, n (%)				< 0.001
Never	7091 (58.6)	3532 (60.8)	3559 (56.6)	
Current	2332 (19.3)	1309 (22.5)	1023 (16.3)	
Former	2669 (22.1)	967 (16.6)	1702 (27.1)	
Sleep hours, n (%)				< 0.001
<8	7086 (55.6)	3476 (60)	3610 (51.9)	
8-9	4606 (36.1)	1944 (33.6)	2662 (38.3)	
>9	1056 (8.3)	372 (6.4)	684 (9.8)	
Age at first sexual intercourse (years), n (%)				< 0.001
Never	504 (5.7)	279 (5.1)	225 (6.8)	
<16	2346 (26.8)	1545 (28.2)	801 (24.4)	
16-17	2411 (27.5)	1551 (28.3)	860 (26.2)	
18-19	1707 (19.5)	1034 (18.9)	673 (20.5)	
>19	1799 (20.5)	1070 (19.5)	729 (22.2)	
No. of sexual intercourse past year, n (%)				0.693
0	198 (3.4)	157 (3.5)	41 (3.2)	
1-11	1626 (27.9)	1256 (27.8)	370 (28.6)	
12-51	1982 (34.0)	1552 (34.3)	430 (33.2)	
52-103	1203 (20.7)	923 (20.4)	280 (21.6)	
104-364	742 (12.7)	585 (12.9)	157 (12.1)	

365 or more	70 (1.2)	53 (1.2)	17 (1.3)	
No. of sex partners during lifetime, n (%)				< 0.001
0	577 (6.6)	327 (6.0)	250 (7.6)	
<=5	4267 (48.5)	2593 (47.2)	1674 (50.7)	
>5	3947 (44.9)	2568 (46.8)	1379 (41.7)	
Illegal substance use, n (%)				0.032
Yes	3706 (52.1)	2906 (53.0)	800 (49.0)	
No	3406 (47.9)	2573 (46.9)	833 (51.0)	
No.of alcohol consumption past year, Median (IQR)	2.0 (1.0, 3.0)	2.0 (1.0, 4.0)	2.0 (0.0, 3.0)	< 0.001
Body mass index (kg/m2), Mean (SD)	25.8 (8.0)	29.3 (7.3)	24.2 (7.7)	< 0.001
Calories consumption (kcal/day), Mean (SD)	1899.1 (821.6)	2141.2 (901.0)	1772.1 (745.8)	< 0.001
Dietary vitamin E intake, Median (IQR)	6.7 (4.6, 9.6)	7.5 (5.2, 10.8)	6.3 (4.4, 9.1)	< 0.001
HPV infection status, n (%)				< 0.001
Negative	3757 (53.4)	3200 (55.1)	557 (45.4)	
Low-risk HPV	1438 (20.4)	1234 (21.2)	204 (16.6)	
High-risk HPV	1842 (26.2)	1375 (23.7)	467 (38.0)	

Abbreviations: SD, standard deviation; No., number; IQR, interquartile range; HPV, human papillomavirus.

Table S2. Sensitivity analysis of association between dietary vitamin E intake and overall HPV infection after excluding participants with extreme calories consumption.

	No.	Crude[a]		Model 1[b]		Model 2[c]	
		OR (95% CI)	*p*-Value	OR (95% CI)	*p*-Value	OR (95% CI)	*p*-Value
**Low-Risk HPV vs. No HPV**							
Vitamin E intake (mg/d)	5723	0.98 (0.97-0.99)	0.004	0.98 (0.97-1.00)	0.008	0.98 (0.96-0.99)	0.005
Q1 (<5.18)	1429	1(Ref)		1(Ref)		1(Ref)	
Q2 (5.18-7.54)	1451	0.94 (0.78-1.13)	0.509	0.96 (0.79-1.16)	0.669	0.94 (0.83-1.06)	0.330
Q3 (7.55-10.82)	1448	0.87 (0.72-1.04)	0.129	0.89 (0.74-1.08)	0.241	0.86 (0.77-0.97)	0.012
Q4 (>10.82)	1395	0.77 (0.64-0.93)	0.007	0.79 (0.65-0.97)	0.022	0.73 (0.66-0.81)	<0.001
Trend test			0.005		0.016		0.011
**High-Risk HPV vs. No HPV**							
Vitamin E intake (mg/d)	5723	0.98 (0.97-0.99)	0.001	0.98 (0.97-0.99)	0.002	0.98 (0.96-0.99)	0.003
Q1 (<5.18)	1429	1(Ref)		1(Ref)		1(Ref)	
Q2 (5.18-7.54)	1451	0.89 (0.74-1.06)	0.184	0.92 (0.77-1.11)	0.402	0.93 (0.82-1.06)	0.270
Q3 (7.55-10.82)	1448	0.78 (0.65-0.93)	0.006	0.80 (0.66-0.97)	0.021	0.80 (0.71-0.89)	<0.001
Q4 (>10.82)	1395	0.74 (0.62-0.89)	0.001	0.77 (0.63-0.93)	0.007	0.74 (0.67-0.82)	<0.001
Trend test			<0.001		0.002		0.007

Abbreviations: HPV, human papillomavirus; Q, quartiles; OR, odds ratio; CI, confidence interval.

[a]No covariates were adjusted in crude model.

[b]Sociodemographic variables (age, sex, race, education, marital status, number of persons in household, and family income) were adjusted in Model 1.

[c]All covariates presented in **Table 2** were adjusted in Model 2.

Table S3. Association between dietary vitamin E intake and overall HPV infection in participants with complete data.

	No.	Crude[a]		Model 1[b]		Model 2[c]	
		OR (95% CI)	*p*-Value	OR (95% CI)	*p*-Value	OR (95% CI)	*p*-Value
**Low-Risk HPV vs. No HPV**							
Vitamin E intake (mg/d)	3556	0.98 (0.97-1.00)	0.012	0.98 (0.97-1)	0.032	0.98 (0.96-0.99)	0.008
Q1 (<5.18)	796	1(Ref)		1(Ref)		1(Ref)	
Q2 (5.18-7.54)	866	1.02 (0.80-1.30)	0.861	1.08 (0.84-1.39)	0.568	1.08 (0.93-1.25)	0.319
Q3 (7.55-10.82)	928	0.99 (0.78-1.25)	0.909	1.05 (0.82-1.34)	0.721	1.06 (0.93-1.22)	0.384
Q4 (>10.82)	966	0.77 (0.60-0.97)	0.030	0.84 (0.65-1.08)	0.174	0.80 (0.70-0.91)	0.001
Trend test			0.023		0.139		0.136
**High-Risk HPV vs. No HPV**							
Vitamin E intake (mg/d)	3556	0.98 (0.96-0.99)	0.001	0.98 (0.97-0.99)	0.003	0.97 (0.96-0.99)	0.001
Q1 (<5.18)	796	1(Ref)		1(Ref)		1(Ref)	
Q2 (5.18-7.54)	866	0.87 (0.70-1.10)	0.242	0.94 (0.74-1.19)	0.613	0.94 (0.81-1.09)	0.397
Q3 (7.55-10.82)	928	0.79 (0.63-0.98)	0.035	0.84 (0.66-1.07)	0.158	0.85 (0.74-0.97)	0.019
Q4 (>10.82)	966	0.65 (0.52-0.81)	<0.001	0.72 (0.56-0.91)	0.006	0.67 (0.59-0.76)	<0.001
Trend test			<0.001		0.003		0.005

Abbreviations: HPV, human papillomavirus; Q, quartiles; OR, odds ratio; CI, confidence interval.

[a]No covariates were adjusted in crude model.

[b]Sociodemographic variables (age, sex, race, education, marital status, number of persons in household, and family income) were adjusted in Model 1.

[c]All covariates presented in **Table 2** were adjusted in Model 2.
